# Reproduction of Distinct *Varroa destructor* Genotypes on Honey Bee Worker Brood

**DOI:** 10.3390/insects10110372

**Published:** 2019-10-25

**Authors:** Wenfeng Li, Cheng Wang, Zachary Y. Huang, Yanping Chen, Richou Han

**Affiliations:** 1 Guangdong Key Laboratory of Animal Conservation and Resource Utilization, Guangdong Public Laboratory of Wild Animal Conservation and Utilization, Guangdong Institute of Applied Biological Resources, Guangzhou 510260, China; wfli.bees@giabr.gd.cn (W.L.); kingtsar@163.com (C.W.); 2 Department of Entomology, Michigan State University, East Lansing, MI 48824, USA; bees@msu.edu; 3 USDA-ARS Bee Research Laboratory, Bldg. 306, BARC-East, Beltsville, MD 20705, USA; judy.chen@ars.usda.gov

**Keywords:** honey bees, *Varroa destructor*, haplotypes, reproduction

## Abstract

Honey bees play important roles in pollination for many crops and wild plants, but have been facing great threats posed by various pathogens and parasites. Among them, *Varroa destructor*, an obligate ectoparasite of honey bees, is considered the most damaging. Within the last century, *V. destructor* shifted from the original host, the Asian honey bee *Apis cerana* to the new host, the European honey bee *A. mellifera*. However, the reproduction of *Varroa* mites, especially of different haplotypes in the two hosts, is still largely unknown. In this study, we first investigated the existing *Varroa* haplotypes in local colonies in southern China, and then compared the reproduction of different haplotypes on the worker brood of both the original and new hosts by artificial inoculation. We confirmed that there are two haplotypes of *V. destructor* in southern China, one is the Korea haplotype and the other is the China haplotype, and the two types parasitized different honey bee species. Although *Varroa* females from *A. mellifera* (Korea haplotype) are able to reproduce on the worker brood of both honey bee species, they showed better reproductive performance in the new host *A. mellifera* with significantly higher fecundity (number of offspring per mother mite) and reproductive rate (number of adult daughters per mother mite), suggesting that this parasite gains higher fitness after host shift. The data further showed that a short stay of *Varroa* females inside the *A. cerana* worker cells decreased their fecundity and especially the reproductive rate in a time-dependent manner, suggesting that the *A. cerana* worker cells may inhibit *Varroa* reproduction. In contrast, *Varroa* mites derived from *A. cerana* colonies (China haplotype) were entirely sterile in *A. mellifera* worker cells during two sequential inoculations, while the control mites from *A. mellifera* colonies (Korea haplotype) reproduced normally. In addition, all the infertile mites were found to defecate on the abdomen of bee pupae. We have revealed that two haplotypes of *V. destructor* exhibit differential reproduction on the worker brood of the original and new host honey bees, providing novel insights into the diversity and complexity of the reproduction of *V. destructor*.

## 1. Introduction

The honey bee is one of the most important economic insects. By providing the essential pollination services for crops and wild plants, honey bees play a vital role in the sustainable development of modern agricultural industries and the global ecosystem, and produce a wide range of benefits for human well-being [1,2]. There is now mounting evidence indicating that the honey bee colony numbers in Western Europe and the US are in decline, which results in a severe pollination crisis and threatens crop production [3,4,5]. Multiple factors have been proposed driving force behind the global honey bee colony losses, where colony-associated parasites and pathogens are specifically emphasized [2,4,6,7]. Among them, it is believed that *Varroa destructor*, an obligate ectoparasite of honey bees, is the most threatening to bee health [8,9].

*V. destructor* had been misclassified to be another honey bee *Varroa* parasite, *V. jacobsoni* Oudemans for decades until 2000 [10]. *V. destructor* originally parasitizes the Asian honey bees, *Apis cerana*, but successfully switched its host to the European honey bees, *A. mellifera* likely in the Far East in the first half of the last century [11]. The infestation of *A. mellifera* colonies by *V. destructor* was initially found in East and South Asia in the 1950s, and then spread to the rest of the world shortly after, excluding Australia [12]. Unlike in its original host, *V. destructor* has posed a deadly threat to the survival of the new host *A. mellifera*, which lacks the resistant mechanisms as seen in *A. cerana*, due to long-term coevolution [13,14]. There are at least seven haplotypes of *V. destructor* identified based on a 458 bp fragment of the mitochondrial *CO-I* gene, including Korea, Japan/Thailand, Nepal, Vietnam, China, China 2, and Sri Lanka haplotypes [10,15], and much greater genetic variation among *V. destructor* (18 haplotypes) has been revealed using the concatenated mtDNA sequences (2700 bp) [16]. However, only two of them, the Korea haplotype and the Japan/Thailand haplotype, have been found to infest the new host *A. mellifera*. Mites of Korea haplotype have globally spread on *A. mellifera*, while the Japan/Thailand haplotype has a more restricted distribution and only been reported in Japan, Thailand, and North and South America [10].

The life cycle of *V. destructor* can be divided into two phases, the phoretic phase and the reproductive phase. In the phoretic phase, *Varroa* females parasitize honey bee adults, and are previously thought to feed on the host hemolymph, but recently found to consume the fat body [17]. Reproduction of *Varroa* mites only happens inside the sealed honey bee brood cells during the reproductive phase [12]. Drone brood cells are more attractive to mites compared to worker brood, but much less available in natural colonies, as they are reared only during swarming season. The ability to reproduce on worker brood emerges as a critical adaption to the new host, remarkably facilitating the development of *Varroa* population and causing greater damage to *A. mellifera* colonies [18]. In fact, *Varroa* mites that infest *A. mellifera* colonies are able to reproduce as effectively on worker brood as on drone brood [12].

In contrast, *V. destructor* has been found to reproduce exclusively on the drone brood and remain sterile on the worker brood of the original host *A. cerana* [12,14,19,20,21,22,23,24,25,26]. This phenomenon is considered crucial for the tolerance of *A. cerana* towards *V. destructor*, and the balance of the host-parasite relationship [14,20]. The high susceptibility of immature workers to *Varroa* infestation has been proposed as another resistant mechanism [27]. However, more and more studies have raised different arguments on this issue. By both direct observations of natural infestation and artificial inoculation, many studies showed that *V. destructor* are capable of reproducing on the worker brood of *A. cerana* [18,20,25,28,29,30]. It should be pointed out that the haplotypes of *Varroa* mites used in the above studies were different. Interestingly, the mites that reproduced on *A. cerana* worker brood were either the Korea haplotype or the Japan/Thailand haplotype, which are found to infest *A. mellifera*; while those that were considered sterile on the *A. cerana* worker brood were neither Korea haplotype nor Japan/Thailand haplotype. On the other hand, *Varroa* mites derived from *A. cerana* colonies in southwestern China and northern Vietnam, which were both identified to be Vietnam haplotype [10,15], were almost infertile in the worker cells of local *A. mellifera* colonies after artificial inoculation [20,25]. These findings suggest that the genetic variation among *Varroa* mites themselves might determine their reproductive capability on worker brood hosts, and mites of different haplotypes show varying reproductive capability on the worker brood of the same honey bee species, but further studies are needed to confirm this.

Anderson and Trueman [10] first reported a new *Varroa* haplotype, namely, China type in *A. cerana* colonies in southern China (Guangzhou, Guangdong Province). This haplotype was later identified in the same area, but in different cities (Zhongshan and Zhuhai, Guangdong Province) [15]. Additionally, *Varroa* mites derived from *A. mellifera* colonies in the same location were exclusively characterized to be Korea haplotype [15]. The availability of various *Varroa* haplotypes makes it possible to simultaneously investigate their reproductive capability on honey bee worker brood. In the current study, we first confirmed the genetic identity of *V. destructor* from local *A. cerana* and *A. mellifera* colonies in Guangzhou, China. We then artificially inoculated the *A. cerana* and *A. mellifera* worker brood with *A. mellifera* derived *Varroa* mites (Korea haplotype), and determined their reproductive outputs. We also sampled *Varroa* mites from *A. cerana* colonies (China haplotype) and inoculated *A. mellifera* worker cells. The reproduction of these mites was compared with controls (using *A. mellifera* derived mites). We found that *A. mellifera* derived *Varroa* mites (Korea haplotype) reproduced on both *A. cerana* and *A. mellifera* worker brood, but had better fecundity on the latter, while *Varroa* mites from *A. cerana* colonies (China haplotype) were completely sterile on *A. mellifera* worker brood in two sequential inoculation experiments. Our findings provide novel information on the reproduction of *V. destructor* in its original and new host. The fact that distinct *Varroa* haplotypes have different reproductive capability and fitness not only improves our understanding of the complexity of *Varroa* reproduction, but also reminds us to monitor the emergence of new *Varroa* haplotypes more closely in both *A. cerana* and *A. mellifera* colonies, since the new mite haplotypes may have better reproductive performance and result in a more severe damage to the honey bee hosts.

## 2. Methods and Materials

### 2.1. Honey Bees and Varroa Mites

*A. mellifera ligustica* (Aml) and *A. cerana cerana* (Acc) colonies were maintained at the experimental apiary of Guangdong Institute of Applied Biological Resources, Guangzhou, China (23.13° N, 113.29° E), where *V. destructor* were collected. The queen-right colonies were fed with fresh syrup and pollen patty accordingly.

For *Varroa* genetic identification, female adults of *V. destructor* were harvested directly from the sealed worker/drone brood of randomly selected colonies. In brief, brood frames were retrieved from the Aml and Acc colonies. Then the caps of sealed brood cells were opened individually, and female adult mites were collected using a soft paintbrush. The collected mites were frozen immediately at −80 °C.

For *Varroa* artificial inoculation, six Aml colonies that were regularly treated with fluvalinate strips to clear *Varroa* mites and free of obvious clinical symptoms of diseases were selected and marked as healthy colonies, and six healthy Acc colonies were picked out and labeled as well. Five extra Aml colonies that were kept purposely without *Varroa* treatment for at least three months were used as *V. destructor* donor colonies. High level of infestation with *V. destructor* is rarely seen in Acc colonies. Only one Acc colony was found to have a low level of *Varroa* infestation, and was used as a *V. destructor* donor colony.

### 2.2. Genetic Identification of Varroa Mites

Total DNA was isolated from individual mites using OMEGA reagent (Omega bio-tec, Guangzhou, China). Mitochondrial *CO-I* gene was amplified from the total DNA by polymerase chain reaction (PCR) assay with the oligo primers: 5′-GG(A/G)GG(A/T)GA(C/T)CC(A/T)ATT(C/T)T(A/T)TATCAAC-3′ (Forward) and 5′-CCTGT(A/T)A(A/T)AATAGCAAATAC-3′ (Reverse) [31]. The PCR amplification was performed in the following conditions—5 min at 94 °C followed by 30 cycles of 1 min at 94 °C, 30 s at 50 °C and 90 s at 72 °C, then a final extension at 72 °C for 5 min. Three to five mites from each colony were used for genetic identification.

The amplified mtDNA *CO-I* fragment was treated with *Xho* I and *Sac* I restriction enzymes respectively, according to the manufacturer’s instructions (Thermo Fisher Scientific, Rockford, IL, USA). The resulting bands were visualized on agarose gel. The PCR product was cut from the agarose gel, purified, and cloned into a pEASY-T vector (TransGen Biotech, Beijing, China), then sequenced using the M13 forward primer (5′-GTAAAACGACGGCCAGT-3′) and reverse primer (5′-AACAGCTATGACCATG-3′) at the Sangon Biotech (Guangzhou, China). Multiple alignments of *Varroa CO-I* gene sequences was performed by using DNAstar software (v 7.1). A phylogenetic tree of 10 *CO-I* gene nucleotide sequences was constructed with Geneious Software (v 11.1.3; Biomatters, Auckland, New Zealand). The tree-building method was UPGMA (unweighted paire group method with arithmetic mean), and the genetic distance model used was Tamura-Nei. This phylogenetic tree was tested with 100 bootstrap replicates.

### 2.3. Preparation of Honey Bee Larvae

The fifth instar worker larvae were identified and prepared according to the method described by previous studies [32,33]. Briefly, for both Aml and Acc colonies, the individual brood frames with uncapped brood were removed out from the healthy colonies and placed with a transparent sheet on the top of the frames. The position and capping status of individual comb cells of a brood frame was marked on the transparent sheet. The brood frames were placed back to the colonies. The next morning, the brood frames were taken out from the colonies and mapped to the transparent sheets. The brood frames with newly-capped brood cells over the past 12 h were identified to contain the fifth instar larvae, and were temporarily kept in an incubator (34 °C, 70% RH).

### 2.4. Collection and Inoculation of Varroa Mites

Female adults of *V. destructor* were collected from newly-emerged bees (1 to 2 day old). In brief, frames with sealed brood from *Varroa* donor colonies were removed and placed into mesh-walled cages individually, and then maintained in an insect growth chamber at 34 ± 1 °C, 55 ± 5% RH for 48 h. *Varroa* mites were collected from bee body using a fine needle and a soft paintbrush, and stored in a glass petri dish with wet filter paper on the bottom. Three to five mites from the sample pools for each inoculation were randomly selected and individually sequenced to confirm their genetic identity, and the results were consistent with the former characterization: Aml colony derived mites were Korea haplotype, while Acc colony derived ones were China haplotype.

All obtained *Varroa* mites were used within 1 h to avoid the loss of activity. They were manually introduced into the fifth instar Aml and Acc worker larvae that were prepared as above. Briefly, the wax cap of a selected worker cell was cut along the inner edge of the cell wall and lifted up to make a small gap using a scalpel. Then one single mite was introduced into the cell through the gap using a soft paintbrush. Lastly, the cap was immediately pushed down and re-sealed using a hot steel needle, and the cell was labeled with a different color. The brood frames were put into plastic frame holders until the whole inoculation was accomplished, and then incubated at 34 °C, 70% RH.

To compare the differences of the reproductive outputs between *Varroa* mites derived from distinct honey bee host species, two successive experiments were conducted.

### 2.5. Experiment I. Reproduction of Varroa Mites Derived from A. mellifera Colonies

For Experiment I, the *V. destructor* mites were collected from *A. mellifera* colonies, and hence, named as ‘*Varroa*-Aml’, which were identified as Korea haplotype. The experiment consisted of four treatment groups: (1) Aml_always. The *Varroa* females were exposed to the fifth instar Aml worker larvae and stayed inside of the Aml brood cells for the whole testing period (0 h of stay inside the Acc worker cells). (2) Transferred_8 h. The *Varroa* females were first introduced to the fifth instar Acc worker larvae and stayed for 8 h (8 h of stay inside the Acc worker cells), and then transferred to the fifth instar Aml worker larvae. (3) Transferred_24 h. The *Varroa* females were first introduced to the fifth instar Acc worker larvae and stayed for 24 h (24 h of stay inside the Acc worker cells), and then transferred to the fifth instar Aml worker larvae. (4) Acc_Always. The *Varroa* mites were introduced to the fifth instar Acc worker larvae and kept inside of the Acc brood cells all the time (maximum duration of stay inside the Acc worker cells). The preparation of the fifth instar honey bee larvae and mite collection and inoculation were performed as described above. All the inoculated frames were kept in an incubator (34 °C, 70% RH), and the newly emerged bees were removed every 12 h to avoid hygienic cleaning of mites.

Every infested worker cell was opened and examined at one day before the expected adult eclosion (eleven and ten days post-inoculation for *A. mellifera* and *A. cerana*, respectively). The reproduction of every mother mite was recorded using the following parameters: Number of offspring per mother mite, number of adult daughters per mother mite, and a fertility rate of mother mites (percentage of the mother mites that produced offspring). We also measured the survival rate of mother mites (percentage of living mother mites). Inoculated cells with the mother mites missing were not included in the data analyses.

There were three replicates for each treatment in the whole study except Transferred_8 h (two replicates), performed in spring and early summer 2018, and spring 2019, respectively. In total the sample size for each treatment was as follows: *n* = 80 for Aml_always, *n* = 52 for transferred_8 h, *n* = 88 for Transferred_24 h, and *n* = 97 for Acc_always.

### 2.6. Experiment II. Reproduction of Varroa Mites Derived from A. cerana Colonies

For Experiment II, the *Varroa* mites were sampled from *A. cerana* colonies and named as ‘*Varroa*-Acc’, which were characterized as China haplotype. Due to the limited availability of *Varroa* mites from Acc colonies, our test only focused on the observation to determine if the mites from Acc colonies were able to reproduce on Aml worker brood as *Varroa*-Aml did. The experiment consisted of two treatments: The first was to inoculate fifth instar Aml worker larvae with *Varroa*-Acc (*n* = 13), and the second was to inoculate fifth instar Aml worker larvae with *Varroa*-Aml (Control, *n* = 19). The inoculation was done as described above. All artificially infested brood frames were incubated at 34 °C, 70% RH, and newly emerged bees were removed every 12 h. Similarly, all treated cells were checked at one day before the expected adult eclosion, and the host appearance was recorded by photos. The same parameters used in Experiment I were employed to report the reproductive outputs of mother mites, and the survival rate was recorded as well. After all these operations, namely, the first infestation, were accomplished, the mother mites were retrieved and transferred immediately to new fifth instar Aml worker larvae, that was the second infestation (*Varroa*-Acc: *n* = 11; *Varroa*-Aml: *n* = 17). The entire experiment was conducted in spring 2019.

### 2.7. Statistical Analyses

For the data from Experiment I, normality of distribution and homogeneity of variance was checked using Shapiro-Wilk test and Levene’s test, respectively, to see if the assumptions of analysis of variance (ANOVA) were met. The number of offspring and adult daughters per mother mite did not meet the assumptions, and were analyzed with nonparametric Kruskal-Wallis test followed by Wilcoxon rank sum test with BH adjustment for *post hoc* comparison. Data of fertility and survival rate of mother mites met with the ANOVA requirements, and then were tested with one-way ANOVA followed by Tukey HSD test for *post hoc* comparisons. For the data from Experiment II, a significant difference was determined by unpaired two-sample Wilcoxon test regarding the data on the number of offspring and adult daughters per mother mite, and Pearson’s Chi-squared test for data of fertility and mortality of mother mites. In all cases, a *p* value of <0.05 was taken to be significant. All statistical analyses were performed in software R v 3.6.0 [34].

## 3. Results

### 3.1. Genetic Difference between Varroa Destructor from Aml and Acc Colonies

Restriction enzyme analyses showed that the *CO-I* gene of Aml-derived mites was only cut by *Xho* I, while the *CO-I* gene of Acc-derived mites was digested by both of *Xho* I and *Sac* I (Figure S1A), which indicated that the *Varroa* mites from *A. mellifera* colonies were Korea haplotype, and those from *A. cerana* colonies were either China or China 2 haplotype.

**Figure 1:**
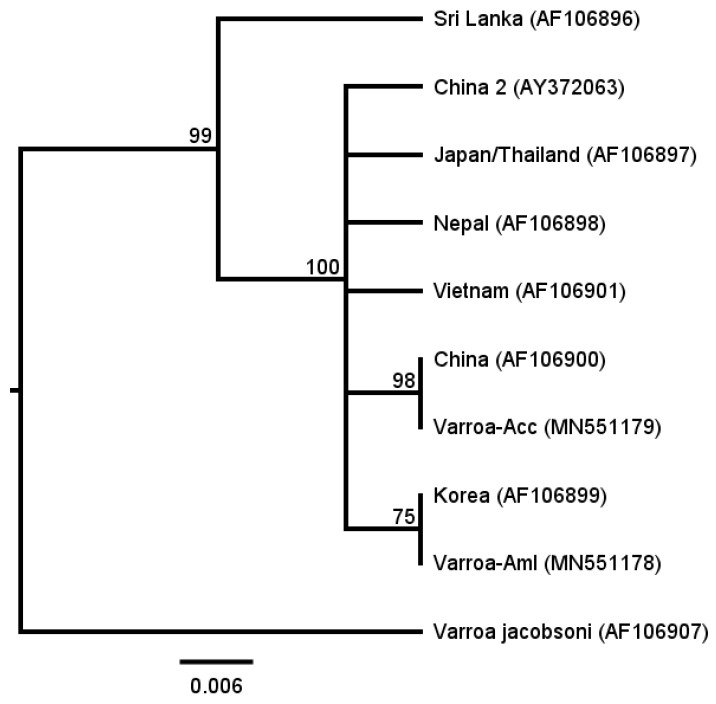
Phylogenetic analysis of *CO-I* gene nucleotide sequences from different *Varroa destructor* haplotypes. *Varroa*-Acc and *Varroa*-Aml stand for the *CO-I* gene sequences of Acc and Aml derived *Varroa* mites, respectively, and *CO-I* gene sequences of seven known *V. destructor* haplotypes and one *V. jacobsoni* haplotype are included (the GenBank accession number are associated). Among them, *V. destructor* mites of Sri Lanka, China, China 2, Nepal, and Vietnam haplotypes were sampled from *A. cerana*, while *V. destructor* mites of Korea and Japan/Thailand haplotypes were collected from both *A. cerana* and *A. mellifera* [10,15]. Bootstrap values (100 replicates) are displayed by the nodes. The genetic distance is drawn to scale. The phylogenetic analysis suggests that the *Varroa* mites collected in the current are either China haplotype (Acc derived) or Korea haplotype (Aml derived).

Sequence analyses of *CO-I* genes further demonstrated the genetic differences between Aml and Acc derived *Varroa* mites (Figure 1 and Figure S1B). The results showed that the *CO-I* gene sequence of Acc-derived *Varroa* mites was identical to that of China haplotype, which is consistent with earlier studies [10,15]. The *CO-I* gene sequence of Aml-derived *Varroa* mites was the same as that of Korea haplotype. Therefore, there were two distinct *V. destructor* haplotypes identified in the local colonies, one was China haplotype, and the other was Korea haplotype.

The sequences of *CO-I* genes were submitted to GenBank and received an accession number MN551178 for Aml-derived *Varroa* mites and MN551179 for Acc-derived *Varroa* mites.

### 3.2. Reproduction of Aml-Derived Varroa Mites on both Acc and Aml Worker Brood

**Figure 2:**
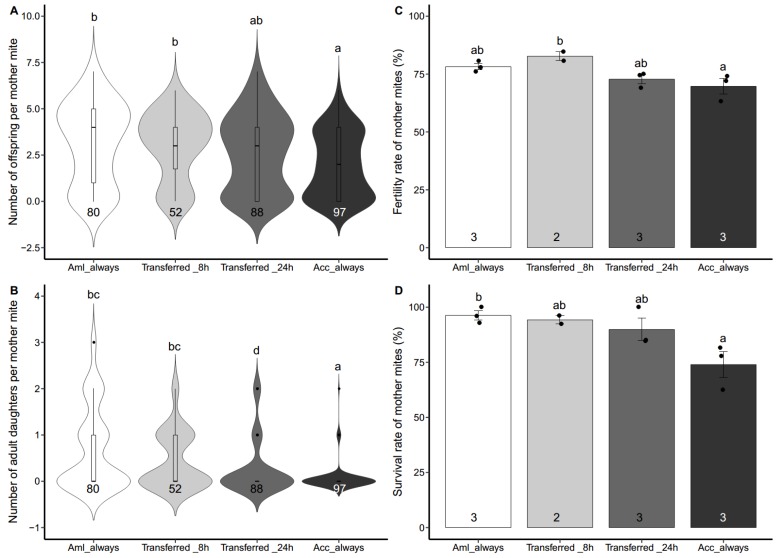
Reproductive outputs of the *Apis mellifera* derived *Varroa* mites on both *A. mellifera* and *A. cerana* worker brood. The mother mites of *V. destructor* were harvested from *A. mellifera* donor colonies, then four different treatments were applied: (1) Mites were inoculated to *A. mellifera ligustica* (Aml) worker brood and then stayed inside all the way (Aml_always); (2) mites were inoculated to Acc worker brood for 8 h, then transferred to Aml worker cells, and kept for the rest of time (Transferred_8 h); (3) mites were inoculated to Acc worker brood for 24 h, then transferred to Aml worker brood, and stayed inside for the rest of time (Transferred_24 h); (4) mites were inoculated to *A. cerana cerana* (Acc) worker brood and then kept inside all the way (Acc_always). (**A**) Number of offspring per mother mite. (**B**) Number of adult daughters per mother mite. Data represent the sum of animals in multiple experiments. (**C**) Fertility rate of mother mites. (**D**) Survival rate of mother mites. Each dot represents data of one independent experiment. For (**A**,**B**), data are presented as median and interquartile range. For (**C**,**D**), data are presented as mean ± SEM, and the numbers of tested mother mites or assays are shown in the bottom of each column. For (**A**,**B**), the statistical significance was analyzed by Kruskal-Wallis test followed by Wilcoxon rank sum test with BH adjustment for *post hoc* analysis. For (**C**,**D**), the significant difference was determined by one-way ANOVA followed by Tukey HSD test for *post hoc* analysis. Lowercase letters above bars denote *post hoc* significance (*p* < 0.05).

We investigated the reproductive performance of *V. destructor* from Aml colonies (Korea haplotype) on the worker brood of both host species. As shown in Figure 2, the *Varroa* mites reproduced differentially on Acc and Aml worker brood, and the reproduction was also affected by the duration of stay inside the Acc worker cells. The fecundity of mother mites among four groups was significantly different (Figure 2A. Kruskal-Wallis test, χ^2^ = 18.125, df = 3, *p* < 0.001). The duration of stay inside the Acc worker cells decreased the number of offspring per mother mite. The *Varroa* females that always stayed in the Acc worker cells reproduced the lowest number of offspring, while those living in the Aml worker cells all the time had the highest number of offspring. Pairwise comparison further revealed the significant differences between groups (Figure 2A. Wilcoxon rank sum test, Acc_always vs. Aml_always: *p* < 0.001; Acc_always vs. Transferred_8 h: *p* = 0.0028).

Similarly, the number of adult daughters differed significantly among the four treatments and decreased with the increase of the duration of stay inside the Acc worker cells (Figure 2B. Kruskal-Wallis test, χ^2^ = 40.684, df = 3, *p* < 0.001). The mother mites that always stayed in the Acc worker cells had the lowest number of mature daughters, and reproduced significantly less adult daughters compared with those from any of the other treatments (Figure 2B. Wilcoxon rank sum test, Acc_always vs. Aml_always: *p* < 0.001; Acc_always vs. Transferred_8 h: *p* < 0.001; Acc_always vs. Transferred_24 h: *p* = 0.0090). Additionally, the number of adult daughters per mother mite of Transferred_24 h was much smaller than that of Aml_always or Transferred_8 h (Figure 2B. Wilcoxon rank sum test, Transferred_24 h vs. Aml_always: *p* = 0.0011; Transferred_24 h vs. Transferred_8 h: *p* = 0.034).

The fertility rate of mother mites presented a small, but significant difference among the four treatments (Figure 2C. one-way ANOVA, *F* (3, 7) = 5.360, *p* = 0.031). Only mites of the Transferred_8 h group had a significantly higher fertility rate than those of Acc_always (Figure 2C. Tukey HSD test, *p* = 0.033). The survival rates of mother mites showed a significant difference among the four treatments (Figure 2D. one-way ANOVA, *F* (3, 7) = 5.289, *p* = 0.032). *post hoc* comparison showed that only mites of Aml_always had a significantly higher survival rate than those of Acc_always (Figure 2D. Tukey HSD test, *p* = 0.032).

### 3.3. Reproduction of Acc-Derived Varroa Mites on Aml Worker Brood

We further examined the reproductive performance of *Varroa* mites from *A. cerana* colonies (China haplotype). Only the reproduction on the Aml worker brood was included because of the limited number of mites obtained. Two sequential infestations were performed and monitored using the same mother mites. In both infestations, all the Acc-derived mother mites survived and had an equal survival rate to the controls (Aml-derived mother mites), but none reproduced, while the control mites reproduced normally (Table 1). More precisely, for the first infestation, reproductive outputs between *Varroa*-Acc and *Varroa*-Aml are statistically different (Table 1. Number of offspring, *p* < 0.001; number of adult daughters, *p* = 0.030; fertility rate, *p* = 0.001). Similarly, for the second infestation reproductive outputs between *Varroa*-Acc and *Varroa*-Aml were also statistically different (Table 1. Number of offspring, *p* = 0.0054; fertility rate, *p* = 0.012), despite of the number of adult daughters showing no difference (*p* = 0.056, Table 1).

We also examined the defecating behavior of both Acc and Aml derived mites on Aml worker cells. All Acc-derived *Varroa* females, which did not reproduce, were found to defecate on the bee abdomen. Most mites from Aml colonies were fertile and found to defecate on the cell walls, while few were sterile, and excreted on pupae body (Figure S2).

## 4. Discussion

In this study, we identified two types (Korea and China haplotype) of *V. destructor* from honey bee colonies located in southern China. We found that these two types of *Varroa* mites were from distinct species of honey bee colonies: The Korea haplotypes only detected in *A. mellifera* colonies, while China haplotype only from *A. cerana* colonies. These results are consistent with the findings reported in the same area two decades ago [15]. Further studies have also described the phenomenon of *Varroa* haplotypes being associated with specific honey bee species (without coexistence) in the same area, such as in southwestern China (China 2 or Vietnam haplotype with *A. cerana* vs. Korea haplotype with *A. mellifera*) and in northern Vietnam (Vietnam haplotype with *A. cerana* vs. Korea haplotype with *A. mellifera*) [15,16].

It seems that different types of *Varroa* mites are naturally separated, even though their living space overlaps, even when, as in this case, they are in the same apiary [35]. The inter-colony mite transmission, however, is feasible and mostly mediated by honey bee drifting and robbing behaviors [36]. Honey bee colonies collapsing from high infestations of *Varroa* mites can act as lures for robber bees, resulting in the elevation of *Varroa* population in the neighboring colonies [36]. Robbing between *A. cerana* and *A. mellifera* colonies is common, especially when the bees are suffering from food shortage. The lack of detection of different haplotypes coexisting in the same host species may be due to some other unknown reasons. We notice that in the areas, such as southern and southwestern China and northern Vietnam only Korea haplotype colonizes *A. mellifera* colonies, while the other types are confined to the original hosts, but it does not mean that these haplotypes are not able, or do not have the opportunities to shift to *A. mellifera*. The details of *Varroa* host shifts are still mysterious, but the sympatric distribution of both honey bee species provides essential opportunities for the parasite to infest the new host [11,12,37]. Monitoring the emergence of new types of *Varroa* in *A. mellifera* colonies seems to be important as they pose a potential threat to the new hosts.

Our results show that *Varroa* mites derived from *A. cerana* and *A. mellifera* colonies are not only distinct haplotypes, but also reproduce differentially on worker brood hosts. By artificial inoculation, we first examined the reproduction of *A. mellifera* derived mites (Korea haplotype) on both *A. cerana* and *A. mellifera* worker brood. The results confirm that in addition to the new host, the mother mites also have the capability to reproduce on the worker brood of the original host *A. cerana*. This has also been demonstrated in previous studies, which were conducted in different parts of China, South Korea, and northern Vietnam, but all involved as Korea haplotype *Varroa* mites [18,20,25,28,29]. Our data revealed that *Varroa* mites produced less offspring and adult daughters in the original host than the new host. It is known that immature workers of *A. cerana* are more susceptible to *Varroa* infestations than those of *A. mellifera*, evidenced by strikingly delayed development and high mortality [27,29], and low tolerance to *Varroa*-derived toxic protein [38]. We suspect that the abnormal development and death of the *A. cerana* worker brood resulted in a shortage of nutrient and energy supplies for the parasite development, contributing to the lower fecundity of mother mites. Obviously, *Varroa* mites fare better following host switch. The better reproductive performance of *V. destructor* on worker brood also accelerates the growth of *Varroa* population, but exaggerates the damage to the new host.

Interestingly, we also found that a short stay of mother mites on the *A. cerana* worker brood decreased their fecundity, especially the number of adult daughters, in a time-dependent manner. This suggests that the *A. cerana* worker cells are able to suppress *Varroa* reproduction. After entering a brood cell, including a fifth instar larva, the mother mite starts to feed on the host fat body [17], certain proteins of which are then transported and stored directly in the ovary of the mite [39]. It is likely that after long-term interaction, the original host has evolved an effective resistant strategy whereby producing toxic and/or nutrient-deficient substances in the hemolymph to suppress reproduction of *Varroa* parasites. On the other hand mite, reproduction can be either triggered or inhibited by the stimuli of a brood cell [12,40]. The oogenesis of *V. destructor* can be induced by the stimuli extracted from the bee larva [41,42,43]. Among them, fatty acid ethyl and methyl esters are most likely to initiate the oogenesis [41]. Meanwhile, inhibitors of *Varroa* reproduction also exist in the host cells. A particular isomer, (Z)-8-heptadecene from infested cells has been shown to decrease the fecundity of *Varroa* females under both laboratory and field conditions [44,45]. We suspect that the original host may suppress the reproduction of *Varroa* mites by regulating the expression of stimuli, and this inhibitive effect can last until reproduction is re-triggered in the new host.

Additionally, the initiation of *Varroa* reproduction was focused during this experimental design, because it is the key step of the whole reproductive process of *Varroa* mites [12,40]. Previous studies have shown that stimuli that promote egg maturation and trigger *Varroa* reproduction are present in capped cells containing larvae and active only within 24 h after cells are sealed [46]. Recently, a study by Frey et al. [41] revealed that the signal that induced *Varroa* oogenesis was produced within 12 h after capping of worker larvae and within 36 h after capping of drone larvae. Once the period had elapsed, another signal began to act in the opposite way, which interrupted the reproduction of *Varroa* mites [41]. This suggests that *Varroa* reproduction is initiated within a short period after the worker cells are capped. For this reason, we made the Transferred_8 h and Transferred_24 h groups to test if a short stay of *Varroa* mites on the original host worker brood inhibits their reproduction.

Likewise, we tested the reproduction of *Varroa* mites from *A. cerana* (China haplotype), but only on the worker brood of new hosts, because of the limited number of mites we could obtain. Since *Varroa* females can reproduce continuously without a phoretic phase [47], we designed an experiment with two sequential inoculations using the same mother mites. Surprisingly, no reproduction was seen in any *Varroa* female in either the first or second infestation, while the control mites from *A. mellifera* colonies (Korea haplotype) still reproduced normally. No matter reproduced or not, both types of *Varroa* mites survived well on the new host, suggesting other living activities, such as feeding and moving were not disturbed. Similar findings were described in previous studies using *Varroa* mites from *A. cerana* (Vietnam haplotype) [20,25]. In addition, Zhou [25] further demonstrated that the mites only reproduced on the drone brood of the original host, suggesting more strict confinement to the reproduction of *Varroa* mites. Despite being infertile on the new host, all the mites from *A. cerana* are found to defecate on the host body. This phenomenon is tightly associated with reproduction of *Varroa* mites, making it a sign of *Varroa* infertility: If the mites defecate on the bee’s body, they will be found to be sterile [48,49]. Normally, the mite families in a cell defecate in the same location on the cell wall, called fecal accumulation site, which acts as a rendezvous platform for mating [50]. The reason why the infertile mites choose to defecate on the bee’s body is still not clear.

Reproduction is the crucial step for the successful parasitism of *V. destructor* in honey bees, and also a main target to suppress the growth of *Varroa* population [12,40]. Our findings further reveal the complexity of *Varroa* reproduction: Different *Varroa* haplotypes reproduce differentially in the original and new host. Considering the economic importance of honey bees and the urgency for the control of *Varroa* mites, our results may help the honey bee conservation from the following aspects in future: (1) To enhance the monitoring of the new *Varroa* haplotypes in both honey bee species; (2) to set up the natural separation between the honey bee populations with different *Varroa* haplotypes to avoid the spillover and/or spillback of the parasites.

## 5. Conclusions

We have confirmed that there are two haplotypes of *V. destructor* in southern China, one is Korea haplotype, and the other is China haplotype. Although their ecological distribution overlaps, the two *Varroa* haplotypes are naturally isolated as they strictly parasitize different honey bee species. Moreover, *Varroa* females from *A. mellifera* colonies have the capability to reproduce on the worker brood of both the original and new hosts; however, better reproductive outputs are obtained on the latter. We further show that the *A. cerana* worker cells might inhibit the reproduction of *Varroa* mites. In contrast, *A. cerana* derived *Varroa* females are entirely sterile in *A. mellifera* worker cells and display abnormal defecation habits. Generally, it is known that infertility on the worker brood is due to a trait of *V. destructor* [20,25], however, despite the fact that they belong to different haplotypes, what exact genetic variations that underpin the differences in reproduction is largely unknown. The genome sequence of *V. destructor* (Korea haplotype) has been already available [51,52], which together with the rapid development of functional genomics, makes the prospect of answering the questions in future.

## Figures and Tables

**Table 1:** Comparison of the reproductive outputs between *Apis mellifera* and *A. cerana* derived *Varroa* mites on *A. mellifera* worker brood.

	First Infestation			Second Infestation		
	*Varroa*-Acc ^a^ (*n* = 13)	*Varroa*-Aml ^b^ (*n* = 19)	Significance ^d^	*Varroa*-Acc (*n* = 11)	*Varroa*-Aml (*n* = 17)	Significance ^d^
Number of offspring per mother mite	0	2.47 ± 0.52 ^c^	*p* < 0.001	0	1.65 ± 0.44 ^c^	*p* = 0.005
Number of adult daughters per mother mite	0	0.63 ± 0.24 ^c^	*p* = 0.030	0	0.29 ± 0.11 ^c^	*p* = 0.056
Fertility rate of mother mites (%)	0	63.16	*p* = 0.001	0	52.94	*p* = 0.012
Survival rate of mother mites (%)	100	89.47	*p* = 0.640	100	100	*p* = 1

^a.^*Varroa*-Acc: *Varroa* mites derived from *A. cerana cerana* colonies. ^b.^ *Varroa*-Aml: *Varroa* mites derived from *A. mellifera ligustica* colonies. ^c.^ Data are presented as mean ± SEM. ^d.^ Significant difference was determined by unpaired two-sample Wilcoxon test for data of number of offspring and adult daughters per mother mite, and Pearson’s Chi-squared test for data of fertility and mortality of mother mites.

## Supplementary Materials

The following are available online at https://www.mdpi.com/2075-4450/10/11/372/s1, Figure S1: Genetic identification of the *Varroa* mites derived from either *Apis mellifera* colonies or *A. cerana* colonies, Figure S2: Defecation of *Varroa* mites on honey bee abdomen.
