# Supplementary material for: Reproduction of Distinct Varroa destructor Genotypes on Honey Bee Worker Brood
Source: Insects. 2019 Oct 25;10(11):372. doi: 10.3390/insects10110372 (PMC6920792; doi:10.3390/insects10110372)
Supplement: Supplementary file 1 [file insects-10-00372-s001.zip › Legends for Figure S1 and S2.docx]

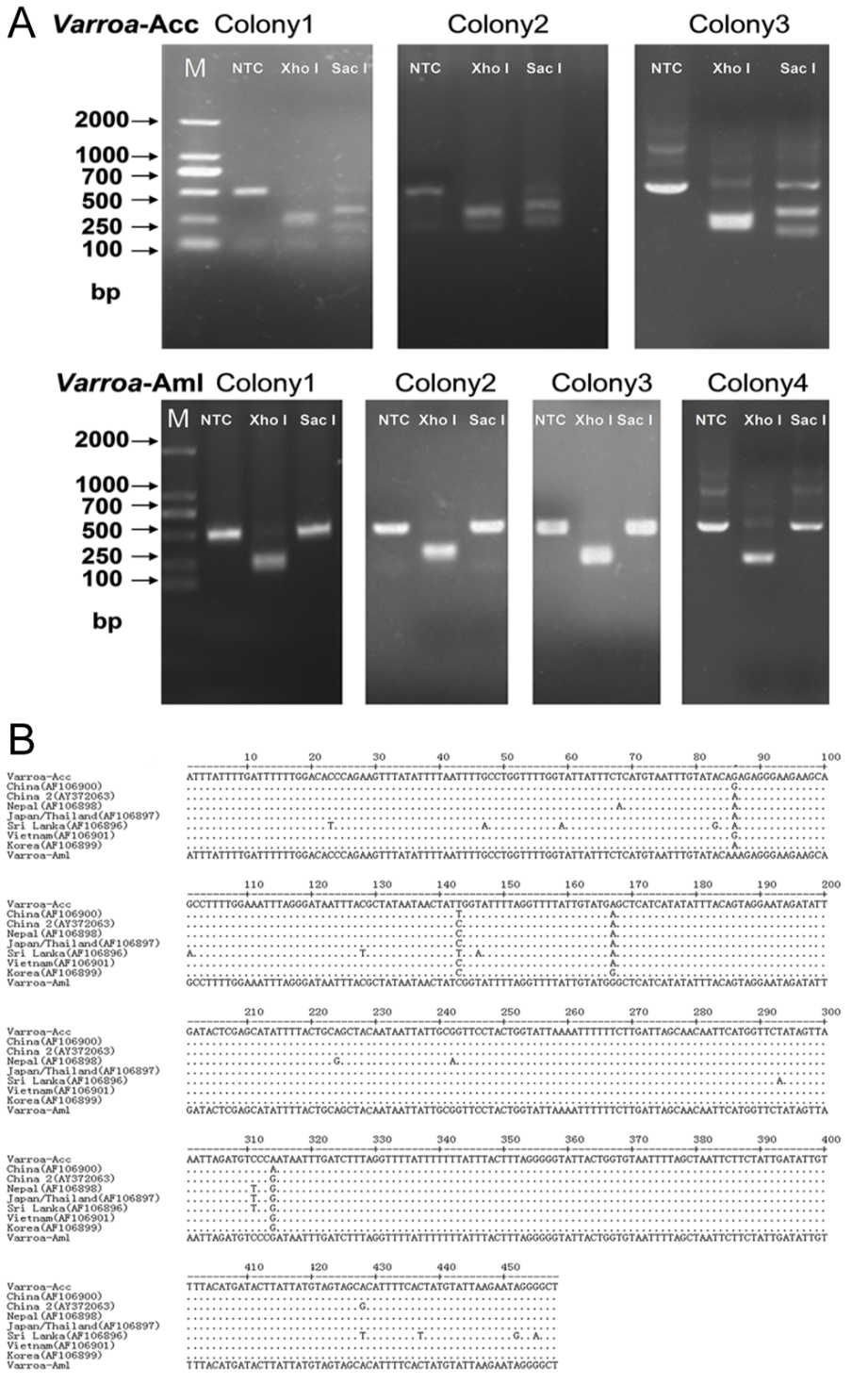


**Figure S1. Genetic identification of the *Varroa* mites derived from either *Apis mellifera* colonies or *A. cerana* colonies. A**, Restriction enzyme analysis. DNA of three to five Varroa mites per colony was individually isolated. CO-I gene was amplified and treated with *Xho* I and *Sac* I restriction enzymes, respectively. A representative gel photo for each colony was displayed. Varroa-Acc indicates the *Varroa* mites derived from *A. cerana cerana* colonies, and Varroa-Aml presents the *Varroa* mites derived from *A. mellifera ligustica* colonies. M: marker (band size is indicated with numbers and arrows on the left); NTC: non-treatment control; Xho I: treated with *Xho I* enzyme; Sac I: treated with *Sac I* enzyme. **B**, Multiple alignment of CO-I gene nucleotide sequences. Varroa-Acc and Varroa-Aml stand for the CO-I gene sequences of Acc and Aml derived Varroa mites, respectively, and CO-I gene sequences of seven known *Varroa* haplotypes were included (the GenBank accession number are associated). The dots mean the same nucleotide with both Varroa-Acc and Varroa-Aml.


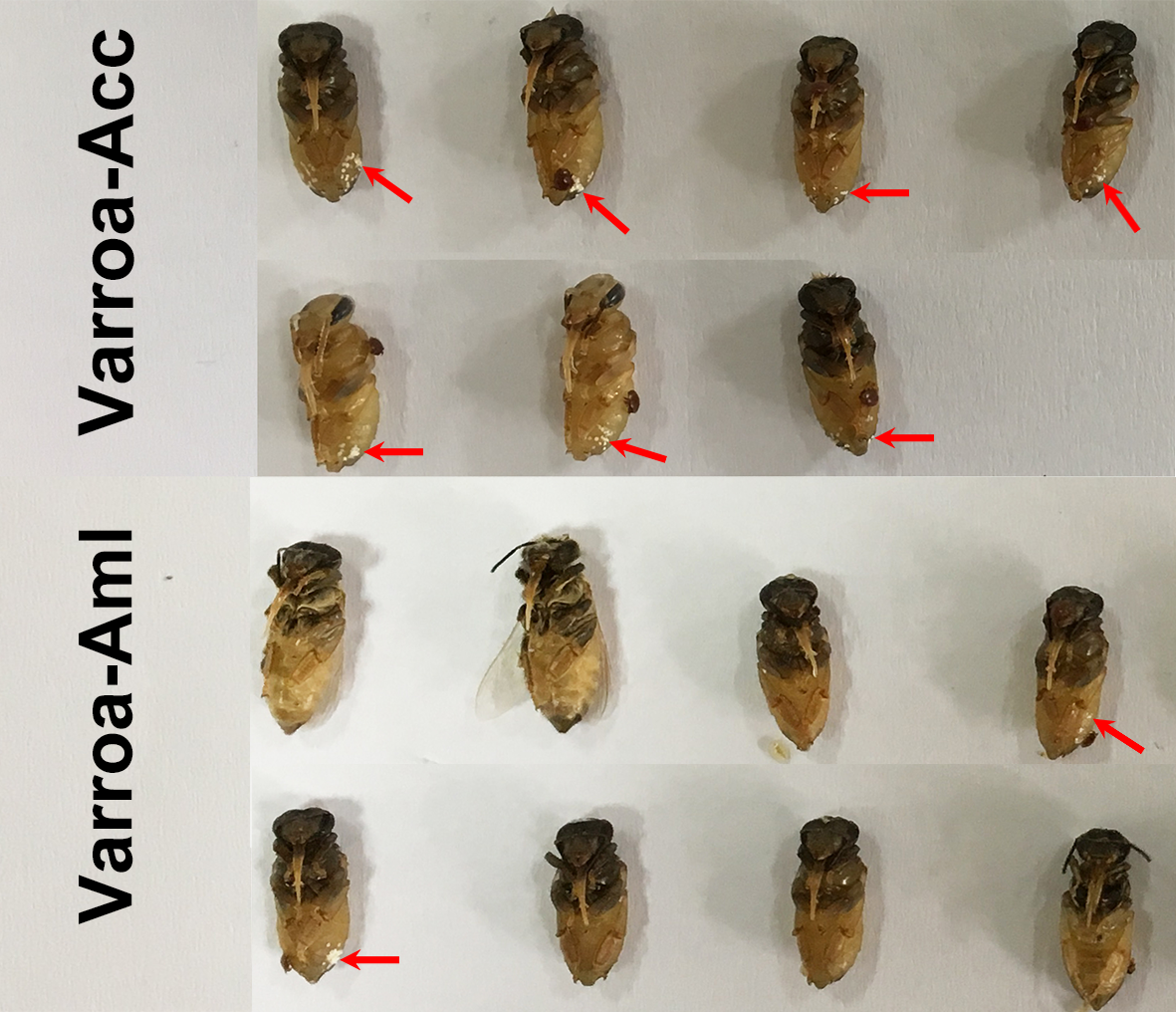


Figure S2. Defecation of *Varroa* mites on honeybee abdomen. The inoculated *Apis mellifera* worker cells with either *Varroa* mites derived from *A. cerana cerana* colonies (Varroa-Acc) or those from *A. mellifera ligustica* colonies (Varroa-Aml) were opened one day before the expected emergence. The pupae inside were removed and laid face up, then photos were taken to show the defecation of *Varroa* mites. Red arrows indicate the *Varroa* mite feces on the ventral side of honeybee abdomen.
