# Supplementary figures and images for: Reproduction of Distinct Varroa destructor Genotypes on Honey Bee Worker Brood
Source: Insects. 2019 Oct 25;10(11):372. doi: 10.3390/insects10110372 (PMC6920792; doi:10.3390/insects10110372)

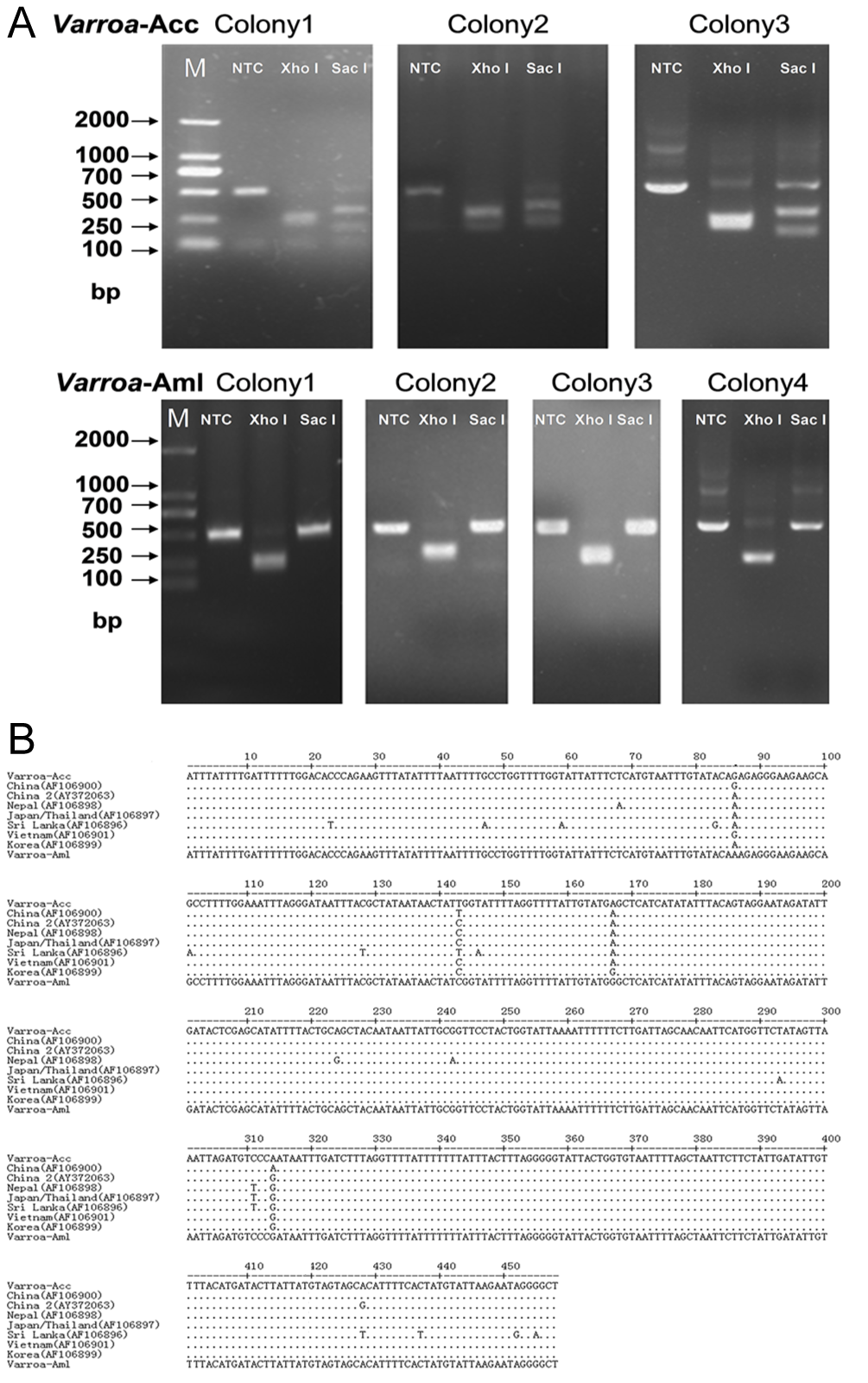

Supplement: Supplementary file 1 [file insects-10-00372-s001.zip › Figure S1.tif]

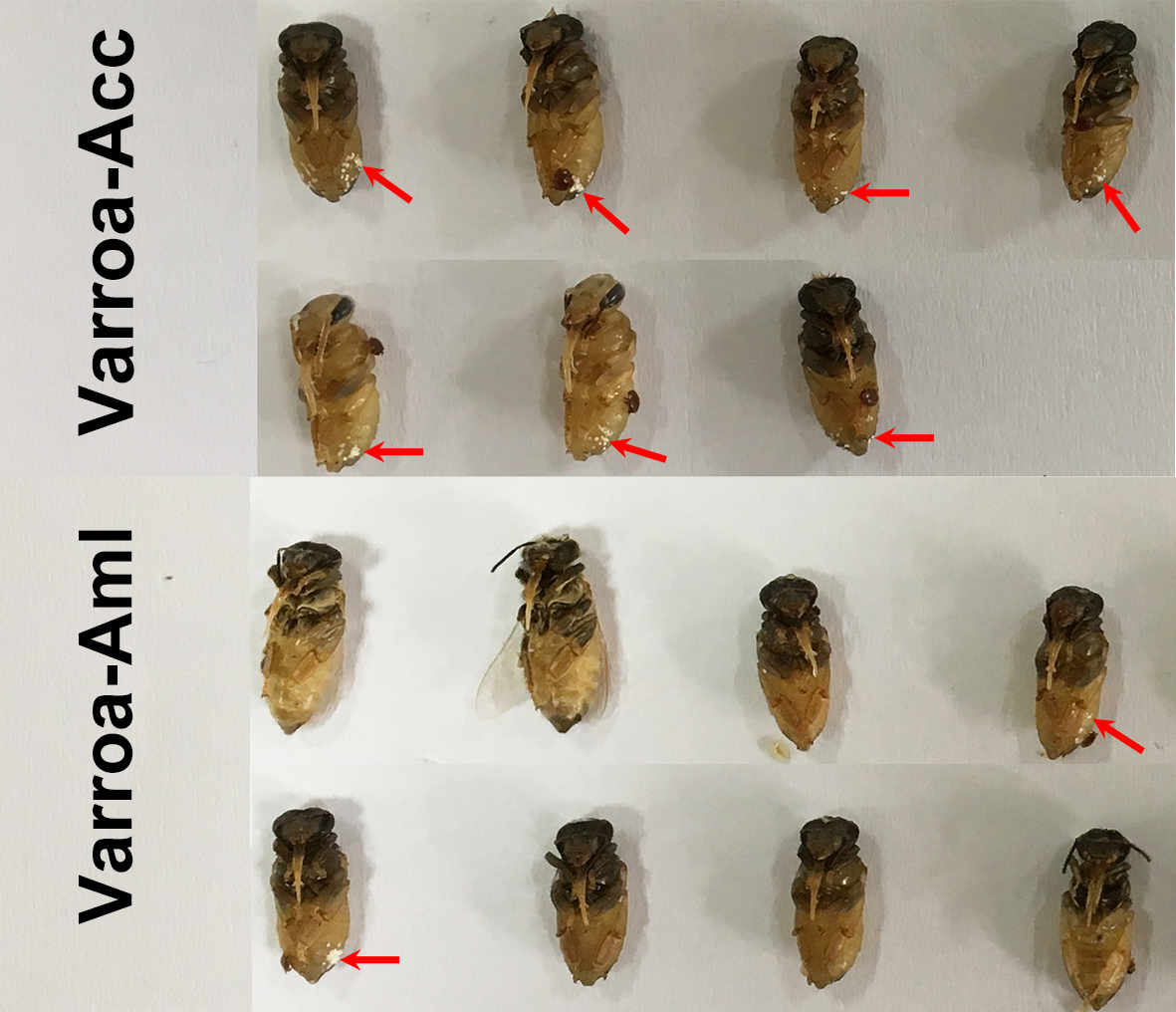

Supplement: Supplementary file 1 [file insects-10-00372-s001.zip › Figure S2.tif]
